# Bridge-Work

**Published:** 1894-12

**Authors:** A. W. Haidle


### Bridge-Work.

BY A. W. HAIDLE, D.D.S.

Read before the Detroit Dental Society, November 12th, 1894.

In what cases is bridge-work advisable and how should the piece be constructed? These questions are of vital importance to the dentist of to-day.

In these closing years of the nineteenth century, when our many colleges are sending out such hordes of young men, nearly all of them fully qualified for performing this class of operation, even the most casual observer must have noticed that it is being used indiscriminately.

The public has become so well informed on this subject that when the patient presents himself with a number of teeth missing, the usual question is put to the dentist, "Can't I have a bridge put in there?" What follows?

The patient is in most cases dismissed in a happy frame of mind, with a piece of bridge-work in his mouth, and minus several dollars.

Why do we find this condition of affairs—this universal cry for bridge-work? Why this servile catering to the demands of the public on the part of the dentist? Is it a healthful condition, and if not how can we best improve it?

In answer to the first question, as to why there is so great a demand for bridge-work? I should say that the demand is a result chiefly of alluring notices in the newspapers, and signs on the windows of some of our practitioners. It is also the result of a feeling of repugnance toward wearing a plate, and a desire for something less cumbersome and more cleanly. This feeling and desire comes quite naturally to those who have already worn a plate, and to others because of a better education among the general public in matters pertaining to the care of the teeth.

To the second question I should make answer by saying, that the dentists accede to the demands of their patients, whether the demands be consistent or otherwise, for several reasons.

Enumerated, they may be stated to be as follows: First, the dentist has before him the prospect of a larger fee for the insertion of bridge-work than for the insertion of an ordinary piece of vulcanite; secondly, if his patient demands bridge-work, he dare not refuse the demand for fear of incurring the displeasure of the patient, and for fear of losing both the work and the patient; and lastly, there is often a desire on the part of the dentist (but mind, I do not say it is not a laudable desire), to display his skill, an ambition to furnish his patients with a higher grade of work, and to become, and to be acknowledged as an expert in this particular branch of dentistry.

Is this a healthful condition, and if not, how can we best improve it?

I should say that it is not a healthful condition. Many bridges are inserted where the conditions plainly indicate a plate; many are improperly constructed; many do not have a sufficiently secure anchorage, and some are not cleanly. We can improve this unhealthy condition by following the dictates of common sense. Let us be more conservative. Let us be certain that a bridge will be the best thing to insert in each particular case before inserting one.

What conditions indicate the insertion of a bridge, and how should the piece be made?

Where one, two or three teeth are missing among the molars and bicuspids, and where the anchorage is secure, one may be put in, provided always that sound teeth are not injured or endangered. If the four incisors have been lost, or any one or two or three of them, a good piece may be made under favorable conditions. Five or six teeth may be attached where there are more than two anchorages, though so extensive a piece of work is rarely advisable. Various other conditions might be mentioned but it is unnecessary.

A number of points however are to be observed in the construction of a bridge.

Always have sufficient and secure anchorage. The attachment of the piece to the anchorage should be strong and durable. Never hesitate if a portion of a sound tooth is to be sacrificed, to sacrifice the whole tooth by cutting away sufficient tooth substance to permit covering of the entire tooth, especially if it be a second bicuspid or one of the molars. In such cases it is better to do this and so protect the tooth from future decay, than to leave it partially exposed by using a band attachment to the then new and favorable conditions for decay.

Where a band attachment is to be made use of, it is always better to make the band at once broad and strong, for with a weak and narrow band the patient will be sure to return and much worry to the operator will result.

Always protect the grinding surfaces of the molars and bicuspids and the cutting edges of the cuspids and incisors with at least one thickness of gold. Never insert a bridge which will prevent the thorough cleansing of the piece itself, and of the surrounding and underlying parts.

In conclusion, unless a bridge is decidedly indicated I should always advise a plate, and advise one having a metallic base, for if the patient can financially afford bridge-work he can equally well afford a plate with one of the noble metals as a base; a gold plate, or one of continuous gum.

